# Outcomes of dietary interventions in the prevention and progression of Parkinson's disease: A literature review

**DOI:** 10.3934/Neuroscience.2024032

**Published:** 2024-12-30

**Authors:** Ubaid Ansari, Alexi Omid, Dawnica Nadora, Jimmy Wen, Arman Omid, Forshing Lui

**Affiliations:** California Northstate University College of Medicine, USA

**Keywords:** Parkinson's disease, neurodegenerative disease, mediterranean diet, vegan diet, carnivore diet, paleolithic diet, ketogenic diet

## Abstract

Parkinson's disease (PD) is a progressive neurodegenerative disorder characterized by motor and non-motor symptoms, primarily due to the degeneration of dopaminergic neurons in the substantia nigra pars compacta (SNpc). Factors contributing to this neuronal degeneration include mitochondrial dysfunction, oxidative stress, and neuronal excitotoxicity. Despite extensive research, the exact etiology of PD remains unclear, with both genetic and environmental factors playing significant roles. Given the increasing prevalence of PD, particularly in aging populations, effective preventive and therapeutic strategies are urgently needed. Emerging research suggests that dietary interventions might offer promising approaches to managing PD progression. This literature review examines various dietary interventions that differ in their composition and mechanisms of action, including the Mediterranean, vegan, carnivore, paleo, and ketogenic diets, and their potential neuroprotective effects. By evaluating the current evidence, this review aims to identify dietary strategies that may improve the quality of life for individuals with PD. Additionally, it explores the underlying mechanisms through which diet may influence PD pathophysiology, thus providing insights into how nutritional modifications can be integrated into holistic management plans for the disease.

## 1. Introduction

Parkinson's disease (PD) is a progressive neurodegenerative disorder with both motor and non-motor symptoms [1]. The hallmark motor symptoms are primarily caused by degeneration of dopaminergic neurons in the substantia nigra pars compacta (SNpc) [1]. Probable factors that contribute to the degeneration of these neurons observed in PD include an impairment of mitochondrial function, a heightened oxidative stress, and an increased neuronal excitotoxicity [2]. The affected neurons typically contain intranuclear eosinophilic inclusions composed of α-synuclein or Lewy bodies. Manifestations of PD encompass motor irregularities such as tremors, rigidity, bradykinesia, and postural instability, alongside cognitive decline, mood disorders, and autonomic dysfunction [1]. Dementia is usually a late discovery in PD. Despite extensive research, the precise etiology of PD remains elusive, with genetic and environmental factors contributing to its pathogenesis. The growing prevalence of PD, particularly in aging populations, underscores the urgency for effective preventive and therapeutic strategies. As the search for effective treatments continues, emerging research suggests that dietary interventions might offer promising avenues to manage the progression of PD. Nutritional approaches, ranging from specific diets such as the Mediterranean and ketogenic diets to the supplementation of individual nutrients such as antioxidants and omega-3 fatty acids, have shown varying degrees of efficacy in clinical and preclinical studies.

The Mediterranean diet (MedD) is rich in fruits, vegetables, whole grains, olive oil, and high-quality proteins such as fish and poultry. It is praised for its high antioxidant and anti-inflammatory content, which may protect against neuronal damage in various neurodegenerative diseases [3]. The vegan diet, which excludes all animal products and focuses entirely on plant-based foods, has been recognized for reducing oxidative stress and inflammation, which are both implicated in the progression of PD [4]. Conversely, the carnivore diet, which solely consists of meat and other animal-derived foods, challenges traditional nutritional guidelines but merits consideration for its unique effects on neuroinflammation and metabolic pathways [5]. The paleo diet is based on the dietary patterns of our Paleolithic ancestors. It includes lean meats, fish, fruits, vegetables, nuts, and seeds, and aims to enhance overall health by avoiding processed foods and grains, thus potentially benefiting brain health [6]. Finally, the ketogenic diet, characterized by a high fat and low carbohydrate intake, has shown promise in various neurological disorders by inducing ketosis. Ketosis is a metabolic state that may offer neuroprotective benefits and symptom relief in PD [7]. Each of these diets presents a distinct approach, with varying mechanisms that may influence the course and symptoms of PD.

This literature review aims to systematically examine the outcomes of different dietary interventions in the prevention and progression of PD. By evaluating the current evidence, this review seeks to identify promising dietary strategies that may offer neuroprotective benefits and improve the quality of life for individuals with PD. Furthermore, this review will discuss the underlying mechanisms through which diet may influence PD pathophysiology, thus providing a comprehensive understanding of how nutritional modifications can be integrated into holistic management plans for PD.

## 2. Review

### 2.1. Mediterranean Diet

MedD is a plant-based dietary approach rich in fruits, vegetables, whole grains, legumes, nuts, and olive oil, with moderate fish, dairy, and wine consumption, while limiting red meat and processed foods. It emphasizes fresh, whole foods and a balanced lifestyle, thus offering numerous health benefits, particularly for its role utilizing antioxidants [8]. Plant-based diets have been demonstrated to reduce oxidative stress and inflammation, which are two important mechanistic links for neurodegenerative disorders, including PD. Oxidative stress promotes PD through α-Synuclein aggregation, and inflammation causes dopaminergic neuronal loss via microglial activation. A recent study published in August 2024 showed that polyphenols, the primary active components of the Mediterranean diet, can support redox balance and offer neuroprotection by activating pro-survival pathways, specifically the hormetic vitagene pathway [9]. Specifically, Calabrese et al. found that mushrooms are strong nutritional neuro-nutrients with a strong activity against neuroinflammation in animal models of traumatic brain injuries or rotenone-induced PD, thus concluding that diet modifications may be an innovative therapeutic intervention in neurodegenerative disorders [9]. Thus, a healthy diet rich in nutrients with antioxidants and anti-inflammatory properties may play an important role in preventing the risk of PD.

A 2017 cross-sectional study by Mischley et al. that involved 1,053 participants found that a diet rich in plants and fish was associated with the lowest PD severity scores [9]. The study revealed that the consumption of fresh vegetables, fruits, nuts, seeds, fish, olive oil, wine, coconut oil, fresh herbs, and spices—all key components of the MedD—was significantly linked to lower rates of disease progression. Additionally, this diet has been associated with a reduced incidence of PD and a later age of diagnosis. A 2012 study by Alcalayan et al. aimed to investigate the association of the Mediterranean diet with PD onset, since previous studies have shown that Alzheimer's disease (AD) can be associated with the diet [10]. The study found that a higher adherence to a Mediterranean-type diet was linked to reduced odds for PD after adjusting for all covariates with the following statistical values: Odds Ratio 0.86; 95% Confidence Interval; and P = .010. Moreover, they reported that a lower adherence to the Mediterranean-type diet score was associated with an earlier PD age at onset (β = 1.09; P = .006). In this study, 257 PD participants and 198 controls were asked to complete the Willett semiquantitative questionnaire, which quantified their diet during the study duration. Additionally, a 2023 longitudinal study by Maraki et al. found that an adherence to the MedD was associated with a lower increase in the probability of developing prodromal PD over time, as well as a reduced incidence of possible prodromal PD, PD, and dementia with Lewy bodies (DLB) among older Mediterranean adults [11]. However, it is worth noting that further research is needed to validate these findings in other populations.

Interestingly, a more recent study published in 2024 showed that a major component of extra virgin olive oil (EVOO), hydroxytyrosol (HT), can interact and regulate S100A9, which is a pro-inflammatory protein known to be involved in neuroinflammation diseases such as AD and PD [12]. This protein accumulates with other proteins such as α-synuclein in PD and Aβ in AD, thus leading to the formation of amyloid plaques that contribute to its neurotoxicity. Leri et al. reported that the formation of HT can reduce the levels of the highly reactive 3,4-dihydroxyphenylacetaldehyde (DOPAL) and the formation of aldehydes such as 4-hydroxynonenal and malondialdehyde, which are biomarkers of oxidative stress. This study suggests the importance of exploring natural compounds, such as HT, as potential effective interventions for complex neurological conditions such as AD and PD.

Conversely, a comparative cross-sectional study that involved 120 PD patients and 50 control patients in Isfahan, Iran, revealed that an adherence to the MedD did not significantly differ between the PD patients and the control group (p > .05) [13]. Additionally, there was no significant association between dietary patterns and PD severity. Diet adherence was assessed by evaluating the participants' dietary intake data using a food frequency questionnaire, while the severity of PD was measured using the Unified Parkinson's Disease Rating Scale (UPDRS). Given the findings from these recent studies, further research is needed to explore how MedD and specific foods within this dietary regimen influence the risk and severity of PD.

### 2.2. Vegan Diet

The vegan diet is exclusively composed of plant-based foods such as fruits, vegetables, beans, grains, nuts, and seeds. Additionally, individuals who follow a vegan diet do not consume meat, dairy, eggs, or honey [14]. This lowered intake of saturated fats leads to reduced total cholesterol and low-density lipoprotein (LDL) cholesterol levels, while also improving the serum glucose control [15]. Many individuals adopt this lifestyle for ethical or environmental reasons, thereby frequently incorporating other lifestyle changes along with dietary adjustments [15]. A vegan diet has attracted interest for its potential to both prevent PD and slow its progression. Being a multifaceted neurodegenerative disorder, PD presents an individual with numerous challenges. However, the significant health benefits associated with a vegan diet make it a compelling option to prevent and manage this debilitating disease. A prospective analysis of the UK Biobank conducted by Tresserra-Rimbau et al. investigated the relationship between plant-based diets and PD incidence among 126,283 participants from the UK Biobank cohort over 11.8 years [16]. Researchers derived three plant-based diet indices: the overall plant-based diet index (PDI), the healthful plant-based diet index (hPDI), and the unhealthful plant-based diet index (uPDI). Using multivariable Cox regression models, they assessed the PD risk across quartiles of these indices. The results indicated that a higher hPDI and an overall PDI were associated with a lower PD risk (22% and 18%, respectively), while a higher uPDI was linked to a 38% increased risk [16]. Specifically, the increased intake of vegetables, nuts, and tea correlated with a reduced PD risk (28%, 31%, and 25%, respectively). The beneficial effects were mainly significant for individuals with a lower polygenic risk score for PD. The study concluded that a healthy plant-based diet, particularly rich in vegetables, nuts, and tea, was associated with a lower risk of developing PD [16].

A vegan diet may also play a significant role in preventing PD by incorporating fruits, vegetables, and whole grains, which are an excellent source of antioxidants and anti-inflammatory compounds [17]. Eating antioxidant-rich functional foods may protect against the oxidation of molecular compounds and protect the body from the effects of free radicals. Free radicals can be produced either through the normal cell metabolism or by an exposure to other external factors such as ultraviolet (UV) radiation or cigarette smoke [17]. Antioxidants have a unique function of donating electrons to disrupt cell oxidation chain reactions, which could irreversibly damage cells [18]. In terms of a vegan diet, diverse antioxidants such as vitamins C and E, polyphenols, and carotenoids work synergistically to prevent the development of potentially harmful reactive oxygen species (ROS) within the cells' mitochondria. Therefore, by reducing the overall oxidative stress, a vegan diet can significantly improve the mitochondrial health, where most antioxidants are produced [18]. Additionally, the consumption of fruits, vegetables, and whole grains is inversely associated with the risk of inflammation. Plant foods contain certain bioactive compounds, specifically carotenoids and flavonoids, which appear to modulate and inhibit inflammatory processes in the human body [19]. A study conducted by Menzel et al. found no significant differences between vegans and omnivores in inflammatory biomarkers such as the high-sensitivity C-reactive protein (hsCRP), interleukin-18 (IL-18), interleukin-1 receptor antagonist (IL-1 RA), intercellular adhesion molecule-1 (ICAM-1), adiponectin, omentin-1, and resistin. However, the duration of a vegan diet was positively correlated with the resistin, IL-18, and IL-1 RA levels [20]. This could suggest that maintaining a vegan diet for extended periods of time can have a positive influence on inflammatory biomarkers in the body; however, further research is needed to evaluate this proposed association.

Another study by McCarty et al. investigated that the risk of developing PD was lower in cultures that adopted a quasi-vegan diet. A quasi-vegan diet is a flexitarian diet that is mostly plant-based but may occasionally include some animal products [21]. Mitochondrial problems in dopaminergic neurons of the SNpc are a key aspect of PD. Parkin, a protein that helps clear out damaged mitochondria and promotes the creation of new ones, plays a protective role. Boosting the Parkin levels in the brain can help to protect against PD, as seen in previous rodent studies. A plant-based diet low in certain proteins may elevate the levels of Parkin and the protective protein PTEN-induced kinase 1 (PINK1) by activating specific cellular pathways [21]. Historically, regions such as East Asia and sub-Saharan Africa, where plant-based diets are common, have shown lower rates of PD. This suggests that such diets might help protect against PD by enhancing the mitochondrial health. Additionally, supplements such as N-acetylcysteine and foods high in spermidine, such as corn, may also support the mitochondrial function and offer protection against PD.

### 2.3. Carnivore Diet

Nutrition has been linked to the risk of developing and managing PD. Notably, dairy products and metabolic syndrome are both risk factors that have been associated with a higher risk of developing PD [22]. A 2018 meta-analysis found that the relative risk associated with metabolic syndrome was positively associated with the total meat intake (1.14, 95% CI: 1.05, 1.23), red meat (1.33, 95% CI: 1.01, 1.74), and processed meat (1.35, 95% CI: 1.18, 1.54), and inversely associated with the white meat intake (0.86, 95% CI: 0.76, 0.97) [23]. Additionally, meat eaters tend to have larger energy intakes, consumption of saturated fatty acids, protein, vitamin B2, B12, zinc, and iodine [24]. The carnivore diet is based around the consumption of animal products, with the elimination or minimal consumption of plant-based foods. The macromolecular composition of this diet resembles the ketogenic and Atkins diet, with low levels of carbohydrates [25],[26]. Similar to other low-carbohydrate diets, low-density lipoproteins (LDL) are markedly elevated compared to high-density lipoproteins (HDL) and triglycerides (TG) [26]. The role and biological mechanisms of cholesterol in the development and management of PD have not been well elucidated. Some studies associate higher LDL and cholesterol levels with a lower risk of PD; however, these findings are indeterminate [27],[28]. Moreover, cholesterol is utilized in neurons for synaptogenesis and is hypothesized to promote the repair of damaged PD neurons [28]. However, the serum cholesterol is unable to penetrate the blood brain barrier (BBB) and therefore is not reflective of the brain cholesterol levels [28]. Another consequence of a low-carbohydrate diet is the increased production of ketone bodies. Ketone bodies have been shown to exhibit neuroprotective effects through the activation of G protein-coupled hydroxycarboxylic acid (HCA) receptors and anti-inflammatory effects via the inhibition of pro-inflammatory cytokines interleukin-1β (IL-1β) and IL-18 [25]. At the mitochondrial level, complex I dysfunction is a common hallmark of PD. It has been speculated that complex I dysfunction is solely sufficient to cause parkinsonism and motor dysfunction [29]. Ketone bodies primarily pass through complex II, thus allowing proper mitochondrial function and energy production [7].

Levodopa is considered the “gold standard” treatment of PD. The protein intake has been shown to affect levodopa absorption in the gastrointestinal tract and the brain via the BBB. As proteins are broken down into amino acids, it competes with levodopa absorption in saturated facilitated large neutral amino acid transporters (LNAA) and can lead to a reduced effect of pharmacological therapy for PD. Notably, the area under the curve (AUC) tends to greatly increase, while the maximum peak concentration (Cmax) and the time to peak absorption (Tmax) have no significant changes, thus indicating no significant effect in the absorption process of levodopa. However, the total amount of levodopa that crosses the BBB is decreased due to changes in LNAA levels [30]. To ameliorate this effect, is it recommended to take levodopa one hour before or two hours after protein-rich meals. Additionally, high-fat and high-calorie meals should be avoided, as they can delay absorption by 2 hours [31]. As PD progresses, the response and effect of levodopa decrease, and the motor function becomes closely related with the rise and fall in the levodopa concentrations. Literature dating as far back as the 1970s has reported that an excess protein intake can play a role in PD progression [30]. Thus, protein intake must be carefully monitored in PD patients to manage motor fluctuations. However, this can also lead to an inadequate total protein intake, thus contributing to decreased muscle quality and function. The recommended dietary allowance (RDA) for protein intake is 0.8 g/kg/day; however, this requirement is well below the recommendation for the elderly, which is between 1.2 and 2.0 g/kg/day [32]. Future studies are required to explore body composition changes, nitrogen balance, and the overall effect on pharmacological therapy between protein intake and PD.

Constipation is another feature of PD and can affect levodopa absorption secondary to small intestinal bacterial overgrowth. This can be managed by consuming adequate levels of fiber and liquids. A high fiber intake can increase the production of short-chain fatty acids via the gut microbiota and can improve the intestinal motility [7]. It has been suggested that the carnivore diet contains fiber and other essential nutrients, though with varying levels depending on the animal product consumed [33]. However, the long-term effects of the carnivore diet on meeting essential and micronutrient needs are still unknown. High levels of meat and fish consumption have been associated with diminished bowel movement frequencies compared to diets that contain larger amounts of plant-based foods [34]. However, a 2021 survey with 2029 participants who consumed a carnivore diet for over six months found that the adverse events were generally low, and constipation was notably reported at 3.1% [26].

However, PD patients are often underweight and malnourished secondary to the underconsumption of foods due to hyposmia and food avoidance (constipation causing GI complaints) [22]. Recent research has also suggested a link between gut microbiome dysregulation and PD via increased systematic and CNS inflammation [35],[36]. In several mouse studies, a low-protein diet (LPD) has been shown to improve motor function by reducing microglial activation, thus lowering proinflammatory cytokine levels and increasing the number of tyrosine hydroxylase-positive neurons. However, this has not been well elucidated in human trials and the PD mice study did not evaluate the effect on levodopa or motor fluctuations [30],[37]. Protein redistribution diets (PRD), which involve restricting daytime protein consumption, have been studied more than LPD and have found improvements in the levodopa sensitivity, disability scores, and adherence at 1-2 years. However, there is an increased prevalence of dyskinesia and weight loss [37]. Both LPD and PRD benefits require more high-quality randomized controlled trials to better elucidate their effects. Cucca et al. investigated the effect of amino acid supplementation across 6 months and found a significantly decreased oxidized glutathione level, albeit without a subsequent decrease in oxidative stress [38]. Importantly, amino acid supplementation did not show any detrimental effects on the neurological, pharmacological, or motor performances.

### 2.4. Paleo Diet

The Paleolithic diet, or paleo diet, is based on the foods likely consumed during the Paleolithic Era. While there is some variation in the specific components of this diet, it generally includes lean meats, seafood, and plant-based foods, including vegetables, fruits, nuts, and seeds, all of which could have been foraged by early humans, depending on their geographic location and climate [6],[39],[40]. A distinguishing characteristic of the paleo diet is the exclusion of dairy products, refined fats, sugars, and processed foods, which became prevalent with the advent of the agricultural and industrial revolutions [6],[40]. It is hypothesized that these dietary changes may have contributed to the rise in chronic diseases due to nutritional deficiencies. Consequently, the paleo diet is considered a potential strategy to reduce the risk of such chronic conditions [6].

Although no currently published studies have investigated the connections between PD and the paleo diet, multiple studies have explored the role of similar diets and food groups on the disease. Similar to the paleo diet, the Mediterranean-DASH Intervention for Neurodegenerative Delay (MIND) diet encourages the consumption of fruits, vegetables, leafy greens, beans, fish, and lean meat while discouraging dairy and processed foods. However, the MIND diet differs in that it includes whole grains and legumes [41]. In a Canadian cross-sectional study, researchers discovered that adhering to the MIND diet was strongly correlated to a later onset of PD by up to 17.4 years in females [42]. Additionally, in a longitudinal study of 706 aging adults, researchers discovered that adhering to the MIND diet was linked to a slower progression and reduced incidence of PD compared to adhering to a MedD [43]. A separate study analyzed the disease progression and dietary components of 1053 individuals with PD via patient-reported outcomes in PD (PRO-PD) and a food frequency questionnaire (FFQ), respectively. Foods associated with a decreased rate of PD progression included fresh fruits, fresh vegetables, nuts and seeds, and non-fried fish. On the other hand, foods associated with an increased rate of PD progression included ice cream, yogurt, and cheese [8].

### 2.5. Ketogenic Diet

The ketogenic diet, characterized by high fat and low carbohydrate intake, may address the bioenergetic deficits in PD [7],[44]. Neurons affected by PD struggle to efficiently utilize glucose for energy production, though they can still utilize ketone bodies such as beta-hydroxybutyrate, which are generated in response to the ketogenic diet [44]. These ketone bodies enable neurons to feed electrons into the mitochondrial respiratory chain at complex II, thus bypassing deficiencies related to the complex I metabolism. Notably, defects in the complex I activity have been observed in the substantia nigra and frontal cortex of individuals with PD. The ketogenic diet's ketones may counteract this defect through a complex II-dependent mechanism, thus enhancing mitochondrial oxidative phosphorylation in the brain [45]–[48].

One study that compared 38 PD patients, 20 on a low-fat diet, and 18 on a ketogenic diet over an eight-week period showed promising results. Both groups showed improvements in all four parts of the UPDRS. However, the ketogenic diet group exhibited greater improvements in the Part 1 scores, which represents non-motor symptoms of PD. Notably, the ketogenic diet group showed relative improvements in cognitive impairment, urinary problems, daytime sleepiness, fatigue, and pain. Notably, since these nonmotor symptoms are least responsive to L-DOPA [44], this study reveals how a ketogenic diet can potentially be used alongside L-DOPA in PD treatment. On the other hand, there were no significant differences in Parts 2 to 4 of the UPDRS scores between the two groups [27]. A similar study had 16 individuals with PD follow a ketogenic diet over a 12-week period. The results showed notable improvements in the UPDRS Part I scores and Parkinson Anxiety Scale (PAS) scores, thus indicating that the ketogenic diet may have potential benefits for non-motor symptoms in PD [49].

In a separate 8-week study, 14 PD patients with mild cognitive impairment were equally divided into a ketogenic diet and a high-carbohydrate diet group. Those in the ketogenic diet group showed greater improvements in lexical access and reduced interference in memory, thus further highlighting the potential cognitive benefits of a ketogenic diet. However, motor function, which was evaluated via the UPDRS Part III scores, remained unchanged [50]. Another recent study investigated the effects of a ketogenic diet on PD via the supplementation of medium-chain triglyceride (MCT) oil [51]. MCT oil is rapidly absorbed and transported to the liver, where it is converted into ketone bodies through a series of enzymatic processes. This accelerates ketogenesis and reduces the latency period required to achieve ketosis [52]. The investigation placed 15 PD patients into a ketogenic diet with MCT supplementation (MCT-KD) and standard diet (SD) groups and evaluated them over a 3-week period. Both groups showed improvements in the Non-Motor Symptom Scale (NMSS), though the MCT-KD group showed greater improvements. Additionally, there was no significant differences in the motor score or mobility between the two groups per the Timed Up and Go (TUG) test or the UPDRS Part 3 [53].

However, a study which highlighted the potential benefits of a ketogenic diet to improve the PD motor symptoms explored its impact on voice quality. The study assigned 68 patients with a voice disorder related to their PD to either the ketogenic diet or the regular diet (RD) group. The participants' Voice Handicap Index (VHI) changes were tested before and three months after their respective diets. The results showed that the ketogenic diet group had an improvement in all mean VHI parameters, thus suggesting that the diet may positively impact voice quality [54].

## 3. Conclusion

This literature review evaluated various dietary interventions and their potential effects on PD prevention and progression. The evidence indicates that certain diets may offer neuroprotective benefits and enhance the quality of life for PD patients. The MedD, which is rich in antioxidants and anti-inflammatory components, shows promise in reducing the PD severity and delaying disease onset. An adherence to the MedD correlates with a slower disease progression and lower PD incidence, although further research is needed across diverse populations. The vegan diet appears beneficial due to its high antioxidant and anti-inflammatory compound bioavailability. However, a variability in inflammatory biomarker responses and a need for more research on the long-term effects of vegan diets in PD are crucial. The carnivore diet shows mixed results. Although it may offer neuroprotective benefits through mechanisms such as ketone production, its impact on PD remains unclear. The potential adverse effects, such as nutritional deficiencies and exacerbated constipation, require further study. While not extensively studied for PD, the paleo diet shares similar benefits with the Mediterranean and MIND diets. However, direct evidence linking the paleo diet to PD is limited and warrants further exploration. The ketogenic diet may address the bioenergetic deficits in PD by improving the mitochondrial function and alleviating non-motor symptoms. While a ketogenic diet could complement conventional therapies, its effects on motor symptoms need further investigation. While dietary modifications hold promise to influence the PD pathophysiology and management, the heterogeneity in the study results underscores the need for more rigorous and standardized research. Future studies should focus on well-designed randomized controlled trials to clarify the effects of various diets on PD and integrate these findings into comprehensive management plans. Understanding the intricate relationship between diet and PD can pave the way for effective nutritional strategies to complement existing treatments and to improve patient outcomes.
